# The Adverse Drug Reactions from Patient Reports in Social Media Project: Five Major Challenges to Overcome to Operationalize Analysis and Efficiently Support Pharmacovigilance Process

**DOI:** 10.2196/resprot.6463

**Published:** 2017-09-21

**Authors:** Cedric Bousquet, Badisse Dahamna, Sylvie Guillemin-Lanne, Stefan J Darmoni, Carole Faviez, Charles Huot, Sandrine Katsahian, Vincent Leroux, Suzanne Pereira, Christophe Richard, Stéphane Schück, Julien Souvignet, Agnès Lillo-Le Louët, Nathalie Texier

**Affiliations:** ^1^ Laboratoire d'Informatique Médicale et d'Ingénieurie des Connaissances en e-Santé, U1142, Institut National de la Santé et de la Recherche Médicale Paris France; ^2^ Service de Santé Publique et de l'Information Médicale Centre Hospitalier Universitaire de Saint Etienne Saint-Etienne France; ^3^ Department of Biomedical Informatics, Rouen University Hospital Rouen France; ^4^ Expert System Paris France; ^5^ Kappa Santé Paris France; ^6^ Unité mixte de recherche 1138, équipe 22, Institut National de la Santé et de la Recherche Médicale Centre de Recherche des Cordeliers Paris France; ^7^ Institut de Santé Urbaine Saint Maurice France; ^8^ Vidal Issy Les Moulineaux France; ^9^ Santeos Paris France; ^10^ Assistance Publique-Hôpitaux de Paris Hôpital Européen Georges Pompidou Centre Régional de Pharmacovigilance Paris France

**Keywords:** pharmacovigilance, social media, big data, natural language processing, medical terminology

## Abstract

**Background:**

Adverse drug reactions (ADRs) are an important cause of morbidity and mortality. Classical Pharmacovigilance process is limited by underreporting which justifies the current interest in new knowledge sources such as social media. The Adverse Drug Reactions from Patient Reports in Social Media (ADR-PRISM) project aims to extract ADRs reported by patients in these media. We identified 5 major challenges to overcome to operationalize the analysis of patient posts: (1) variable quality of information on social media, (2) guarantee of data privacy, (3) response to pharmacovigilance expert expectations, (4) identification of relevant information within Web pages, and (5) robust and evolutive architecture.

**Objective:**

This article aims to describe the current state of advancement of the ADR-PRISM project by focusing on the solutions we have chosen to address these 5 major challenges.

**Methods:**

In this article, we propose methods and describe the advancement of this project on several aspects: (1) a quality driven approach for selecting relevant social media for the extraction of knowledge on potential ADRs, (2) an assessment of ethical issues and French regulation for the analysis of data on social media, (3) an analysis of pharmacovigilance expert requirements when reviewing patient posts on the Internet, (4) an extraction method based on natural language processing, pattern based matching, and selection of relevant medical concepts in reference terminologies, and (5) specifications of a component-based architecture for the monitoring system.

**Results:**

Considering the 5 major challenges, we (1) selected a set of 21 validated criteria for selecting social media to support the extraction of potential ADRs, (2) proposed solutions to guarantee data privacy of patients posting on Internet, (3) took into account pharmacovigilance expert requirements with use case diagrams and scenarios, (4) built domain-specific knowledge resources embeding a lexicon, morphological rules, context rules, semantic rules, syntactic rules, and post-analysis processing, and (5) proposed a component-based architecture that allows storage of big data and accessibility to third-party applications through Web services.

**Conclusions:**

We demonstrated the feasibility of implementing a component-based architecture that allows collection of patient posts on the Internet, near real-time processing of those posts including annotation, and storage in big data structures. In the next steps, we will evaluate the posts identified by the system in social media to clarify the interest and relevance of such approach to improve conventional pharmacovigilance processes based on spontaneous reporting.

## Introduction

Adverse drug reactions (ADR) are among the most frequent causes of death in industrialized countries [1]. For example, in the Netherlands, ADRs correspond to 140,000 hospital stays per year, which is more than the number of stays for myocardial infarction, and from 10,000 to 30,000 deaths per year are related to ADRs [2]. Due to methodological and patient selection limitations, clinical trials are not designed to detect all ADRs, requiring postmarketing surveillance or pharmacovigilance systems.

Pharmacovigilance is defined as “the science and activities relating to the detection, assessment, understanding, and prevention of adverse effects or any other drug-related problem” [3]. Pharmacovigilance therefore aims to alert about the potential risks of a drug: the detection or generation of a signal. According to Bégaud et al [4], “on average, no more than 5% of serious ADRs are actually reported.” Underreporting by health care professionals is therefore a limiting factor for the efficiency of pharmacovigilance processes. The collection of data directly from patients has been organized by the health authorities of several countries in the past. Studies have shown that some reports from patients can be of similar quality compared to health professionals [5], but reporting by patients remains limited due to lack of awareness of the reporting system.

In the era of Web 2.0, online social networking applications have become very popular, allowing users to communicate, interact, and share worldwide. These social media bridge the geographical and social gap between people, enabling them to share similar experiences, facilitating what is difficult in the real world. Patients using social media have grouped into communities to share a wide variety of personal medical experiences, including use of medicines and adverse reactions. The interactions between patients take many forms, including social networks, blogs, microblogs and discussion boards, emails, and chats, which are potential sources to explore. Extraction of such data and its integration in pharmacovigilance processes face 5 major challenges as depicted in Table 1.

Although research teams have already performed several experiments to extract knowledge from Web forums [6-8], the process is still not operably exploited by most pharmacovigilance teams from drug regulatory authorities and the pharmaceutical industry [9]. We assume the reason is that much effort has been devoted to identifying relevant information within patient posts, but supplementary developments are still required to build specific solutions for the other challenges.

The Adverse Drug Reactions from Patient Reports in Social Media (ADR-PRISM) project aims to develop a method to operationalize the analysis of patient posts in social media. This paper describes the current state of advancement of the ADR-PRISM project by focusing on the solutions we have chosen to respond to the 5 major challenges.

**Table 1:** Major challenges related to exploiting patient posts in pharmacovigilance.

Question	Challenge	Response? Process? Solution?
How to manage social media quality?	Variable quality of information on social media	Identify social media that present high-quality content regarding relevance and completeness of information for pharmacovigilance purposes
How to manage data privacy?	Guarantee of data privacy	Take into account data privacy of patients posting on the Internet
How to deal with pharmacovigilance main objectives?	Response to pharmacovigilance expert expectations	Identify the optimal framework where analysis of patient posts can usefully complement usual pharmacovigilance processes
How to identify relevant information within patient posts?	Identification and processing of relevant information (eg, drugs and adverse reactions) within Web pages	Extract, process, and render relevant information on drugs and their adverse reactions
How to manage scalability related to big data collection in social media?	Robust and evolutive architecture	Take into account the evolution of the platform and the high quantity of data available on the Internet that requires specific methods for big data collection and storage

**Table 2:** Proposed methods for meeting the 5 major challenges.

Challenge	Proposed method
Variable quality of information on social media	Design a scoring method that allows selection of high-quality social media
Guarantee of data privacy	Design a technical solution based on data minimization and access restriction that guarantees data privacy of patient posting on social media
Response to pharmacovigilance expert expectations	Study the pharmacovigilance expert requirements and formalize them in use case diagrams and usage scenarios
Identification and processing of relevant information (eg, drugs and adverse drug reactions) within Web pages	Enforce best practices based on specialized dictionaries, pattern-based matching, and natural language processing to detect drugs and their adverse reactions in patient posts
Robust and evolutive architecture	Build a component-based architecture that allows storage of big data and accessibility to third-party applications through Web services

## Methods

### Overcoming the Five Major Challenges

In order to overcome the 5 major challenges, we propose to apply the methods depicted in Table 2.

### Variable Quality of Information on Social Media

In order to carry out our study about the quality of information on social media, we first sought to identify potential sources of interest. An initial analysis was conducted exploring the functioning and content of a selected sample of websites as well as the behavior and interactions of their users [10].

To identify relevant forums for the project (ie, providing messages about drug use and safety), we conducted research through search engines such as Google with the terms “network,” “forum,” “health,” “patient,” and “medicine,” and 11 sites were identified.

In addition, to identify specialized forums about a disease or group of diseases, for example, we used the *Catalogue et Index des Sites Médicaux de langue Française* (CISMeF) website [11,12], a catalog and index of French language health resources on the Internet that conducts appraisals of the websites of patient associations. These websites were systematically covered by a medical librarian to identify forums, and 17 websites were identified. Forums strictly reserved for members or forums with very little activity (fewer than 10 messages in the current month) were excluded.

We identified, reviewed, and rated the websites according to 3 major criteria (number of visits, popularity of the website, and number of health and drug therapy-related posts) which we estimated using the following methods:

- Collection of total posts if it was indicated
- The website 1001forum [13], one of the most important French forums for indexers, to estimate the number of messages posted per day on each forum
- Google, Alexa [14], and Yoovi [15] to estimate the popularity, traffic, and overall activity of the forum

Finally, for all selected and specialized forums, a pharmacist checked the relevance of each mention of drugs corresponding to the theme of the forum.

A state-of-the-art search of the existing methodologies to assess the quality of the websites was carried out. This led to the identification of the net scoring [16] method, among others [17], which enables the assessment of health websites. This tool, designed for the evaluation of websites but not specifically for forum evaluation, was used to perform a detailed analysis of 7 health websites and their forums among the sites initially identified in order to adapt the criteria of the net scoring grid to the evaluation of social media.

The evaluation of Twitter and Facebook as sources is currently being addressed, and their specific formats (eg, limitation of the number of digits to 140 for Twitter) necessitate a more specific analysis. Tweets are more difficult to interpret than posts in other social media that usually contain more information, but tweets have the advantage of being available in larger volumes. While the contribution of Twitter to pharmacovigilance has already been extensively evaluated, for example, by Bian et al [18], who concluded that “daily-life social networking data could help early detection of important patient safety issues,” little work has been performed with Facebook, which requires further investigation.

### Guarantee of Data Privacy

ADR-PRISM established an ethics committee comprised of a medical doctor specializing in pharmacovigilance, an expert in the management of data privacy, and an epidemiologist to support the consortium in defining the policy for access, data recording, and respect for the privacy of personal information of patients describing an adverse effect on social media. The committee worked on a solution that was ethically acceptable and consistent from a legal point of view.

In parallel, a working group was created and studied the different possibilities for complying with the ethical and confidential aspects of data collection by organizing brainstorming sessions, participating in working groups and conferences on this theme, and consulting the outside advice of a lawyer specializing in the matter. The working group held several meetings with the expert in data privacy from the ethics committee.

### Response to Pharmacovigilance Expert Expectations

We defined several use cases after a compilation and analysis of end-user requirements for the future system. Use cases have been written after thorough analysis of the context of the monitoring of health products, an analysis of foreseen end users of the platform, and the uses they may have of the tool. See Table 3 for the definitions of the concepts of use case diagrams and usage scenarios.

**Table 3:** Definitions of use case diagram and usage scenario.

Definition		Example
**Use case diagram**			
	Unified Modeling Language provides use case diagrams in order to specify and describe use cases. These are cases of the system (ie, a description of how the user interacts with the system). Use cases are generally organized in steps, which can be considered smaller usage components (and therefore can be described as discrete use cases).	An example of use case is the selection of a drug and/or an adverse reaction upon interaction with a form displayed by the system. Another use case is to read posts from social media for this selection.
**Usage scenario**			
	Usage scenarios are circumstances in which a user will interact with the system. Multiple usage scenarios can be defined that can correspond to a single use case.	Making a pharmacovigilance survey is a usage scenario. Indeed, we can decompose the survey into smaller components to look for similar case reports in the national pharmacovigilance database, make a literature search, and perform a search in social media.

### Identification and Processing of Relevant Information Within Web Pages

Information extraction is the process of extracting and analyzing information in order to discover buried knowledge, leading to intelligence from large volume of unstructured text content. Our extraction process involves several subtasks: (1) text preprocessing, including text formatting tasks, (2) morphosyntactic tagging, performed by the XeLDA (Naver Labs Corp) tagger, which identifies the language, splits the text into sentences and words, and attaches to them their lemma and part of speech category, (3) extraction rules compiled in semantic components, which model the relevant information to extract, and (4) postprocessing, to normalize the information extracted.

All analysis steps are packaged in components that build a Skill Cartridge (Expert System France SA). It can be imagined as a cascade of extraction modules that condense the textual data into meaning. The analysis is based on finite state technology [19].

Skill Cartridges are domain-specific knowledge resources that may embed lexicon, morphological rules, context rules, semantic rules, syntactic rules, and postanalysis processing. They are plugged into the Expert System Luxid (Expert System France SA) extraction server to perform annotations. The processing unit is the sentence. The information to be extracted by the Skill Cartridge is modeled according to the Luxid Data Model, which is made up of Luxid Objects. A Luxid Object is qualified by a type, either entity, relationship, or structure. Objects of the same type share the same behavior, meaning, and attributes. A type may have a parent type from which it inherits meaning, attributes, and behavior. A type is represented by the complete hierarchy like /entity/company or /relationship/biomedical/ activation. Entities can either stand alone or be related to each other by a relationship (which in general describes an action).

The Skill Cartridges use multilanguage dictionaries that assign concepts to words and phrases called entities. Each entity, in addition to its preferred label and its variants, may also have attributes. These are fields associated with the concept that contain additional information about the concept. For example, a concept “disease” may have the attribute “course” whose value is “acute.” All these entities are stored in the form of a concept tree. Some concepts have parents and/or children (eg, one can see the concept “neuropathy” as a child of the concept “neurological disease”).

Extraction rules, driven by targeted results, build relationships between defined concepts from the low level to a higher level. The extraction rules process consists in several read-outs, where the analyzed text is successively retagged. During each read-out, the tagged text is replaced by the corresponding concept. During successive read-outs, the extraction server does not see the text but the concepts. Skill Cartridges are used to annotate the messages using medical terminologies that detect a broad medical vocabulary and discern the concepts and semantic relationships related to drugs, associated adverse events, and all additional data that can be attached as the date, location, patient characteristics (age, gender, dosage, etc), characteristics of the medication (dose, frequency, duration, etc), and characteristics of the post (name of the forum, name of the thread, etc).

A working group has been created to develop a process to analyze data and extract some pharmacovigilance information and has identified 3 medical terminologies from the HeTOP terminology server (Health Terminology/Ontology Portal) [20,21] and data from the Vidal Drug Database for the project. The identified terminologies are shown in Table 4.

**Table 4:** Medical terminologies selected for feeding the dictionary for the data extraction task.

Short name	Long name	Content	Language	Number of concepts	Example	Reference
ATC	Anatomical Therapeutic Chemical Classification	Drugs classes and substances	FR/EN	4717	http://www.chu-rouen.fr/cismef/skos#ATC_CD_A/ anabolic agents for systemic use/ anabolic steroids/androstan derivatives/ androstanolone	[22]
MedDRA	Medical Dictionary for Regulatory Activities	Adverse drug reactions	FR/EN	74,413	MedDRA top tree/cardiac disorders/heart failures/cardiac failure/cardiac insufficiency	[23]
Racine Pharma	Roots of the pharmaceutical products	Short names of drugs	FR	5164	Ascorbic acid	manually produced
Vidal Drug Database	Drug database sold by the Vidal company	All the regulatory information about drugs	FR	40,227	XVII congenital malformations and chromosomal abnormalities/ Q00-Q07 congenital malformations of the nervous system/ Q00.0 anencephaly	[24]

In the ADR-PRISM project context, the Anatomical Therapeutic Chemical (ATC) classification enables drugs class and substance entities extraction. Racine Pharma enables extraction of common drug names and abbreviations used by patients. It is a terminology developed by CISMeF from a file of French brand names where “racine” is the French word for “root.” A root corresponds to its brand name label without its strength or form or the name of the company in the case of a generic drug. This terminology is revised monthly according to new updates to the French public drug database. New potential roots are extracted by an informatics process and presented to a pharmacist for curation. Several roots are linked to their ATC code. This terminology is not currently available in open access. The Medical Dictionary for Regulatory Activities (MedDRA) identifies ADRs. Vidal data contains all the regulatory information about drugs (names, indications, contraindications, expected adverse reactions, dosage, posology, interactions, precautions, etc).

### Robust and Evolutive Architecture

The method used to define the system architecture was based on the information system architectures part of the Open Group Architecture Framework. After having defined inputs and outputs provided by each partner of the ADR-PRISM consortium, data and application architecture have been combined to propose a service-oriented architecture. Regarding the storage software, a study comparing several solutions was conducted. Different big data storage technologies have been subject to the project constraints concerning relational or object-oriented database management system as well as non–structured query language solutions: columns oriented, documents oriented, key/value, graphs oriented, in-memory data grids, and triplestores.

The first prototype of the ADR-PRISM project is based on Expert System Luxid repository technology, in which both posts and their indexing metadata (structural metadata and semantic annotations coming from the annotation service) are stored in order to support the search and browse functionalities from Luxid: (1) keywords (full text search), (2) concepts (terms from the thesauri), and (3) relations linking the concepts.

## Results

### Variable Quality of Information on Social Media

The use of the net scoring tool, composed of 46 criteria, in the evaluation of Internet social media has been studied by Katsahian et al [10]. The results showed that the net scoring tool needed to be adapted, clarified, and simplified in order to obtain the most suitable and functional grid. The least relevant criteria were eliminated or grouped into new and more general criteria. The final resulting grid was composed of 21 equally weighted criteria (see Multimedia Appendix 1).

### Guarantee of Data Privacy

The positioning of the project in terms of data processing, collection, and ethical compliance is complex in terms of current regulations and their evolution in the near future. In France, the Data Protection Act of January 6, 1978 (*Loi Informatique et Libertés*), applies as soon as there is automatic or nonautomatic data processing involving personal health data. Article 2 of the law defines personal health data as “any information relating to a physical person who is or can be identified, *directly* or *indirectly*, by *reference* to an *identification number* or to *one* or *more factors specific* to them” [25] **.**

Article 8 of the law specifies that “the collection and processing of personal data that reveals, directly or indirectly, the racial and ethnic origins...of persons, or which concern their health or sexual life, is prohibited.” This article also defines exceptions to this law, for example, for “processing that relates to personal data that the concerned person has made public” or if the personal data are subject to an anonymization procedure which the French data protection authority, *Commission Nationale de l'Informatique et des Libertés* (CNIL), has earlier approved. In that case, the CNIL provides a specific procedure for processing in the scope of conventional pharmacovigilance (number AU-013 unique authorization of the CNIL).

One of the specificities of the ADR-PRISM project lies in the fact that its purposes and the data processing natively refer to several legal and sometimes conflicting environments. Indeed, the analysis of social media to identify ADRs requires at least the following actions to proceed:

- Identification of data types that will be used (personal data, health data)
- Qualification of the data processing to be carried out (collection, treatment, preservation and storage, etc)
- An inventory of texts and regulations governing the operated data processing (French and European, including the decree on health care data hosting, etc)
- Obtaining the necessary authorizations

To respond to this complexity we have chosen an approach that involves:

- Breaking the global treatment related to the ADR-PRISM project into several treatment units that will then be positioned in a known and mastered legal framework
- Articulating the treatment units in order to match them to the global treatment

This approach, subject to the approval of the CNIL, led to the identification of 2 different treatment units:

- Data collection from forums. Data collected are considered as indirectly identifying data (as the pseudonym is collected). Data considered at this stage are the copy of data found on public forums and haven’t been enriched by processing yet.
- Data processing and enrichment. Data are enriched by text mining analyses. Health data emerges at this stage from raw data.

This process relies on the creation of 2 different accesses to the database that will be granted to minimize the risk of reidentification:

- Regular access to the database through a process of data minimization that doesn’t allow access to the verbatim text of the post, the author’s pseudonym, and the name of the forum
- Restrictive access to the posts (cleaned by algorithms aiming to remove all directly identifying data before storage, such as email, telephone number, or addresses), the pseudonym, and the forum

This second access allows the treatment covered by the processing of personal data and data processing steps related to pharmacovigilance requirements (defined by number AU-013 single authorization of the CNIL). These include preserving the possibility of making contact with the author of the message describing the adverse reaction (via its account) to investigate the case report and if necessary to intervene for a medical emergency. This second access is the most sensitive, which explains why it benefits from the highest modalities of information security.

We have completed the process of computer data security with organizational procedures. In particular, we established a limited list of users allowed to benefit from the second restrictive access. This list is controlled by a specific procedure established by the ethics committee and, in exceptional cases, additional special authorizations may be granted after study of the application by the ethics committee.

### Response to Pharmacovigilance Expert Expectations

While the usage scenarios are numerous and depend on the monitored product or type of implementation monitoring method, the use cases related to the platform are relatively similar regardless of the context. For the user, the main usage scenario to use the ADR-PRISM platform will be to:

- Consult cases
- Filter the cases or make queries
- View results
- Select products or ADRs for active surveillance (manage alerts)
- Follow these and see new information on these products
- Be alerted of new cases
- Extract the results (data stream or aggregated tables)
- Identify new pharmacovigilance signals

**Figure 1:**
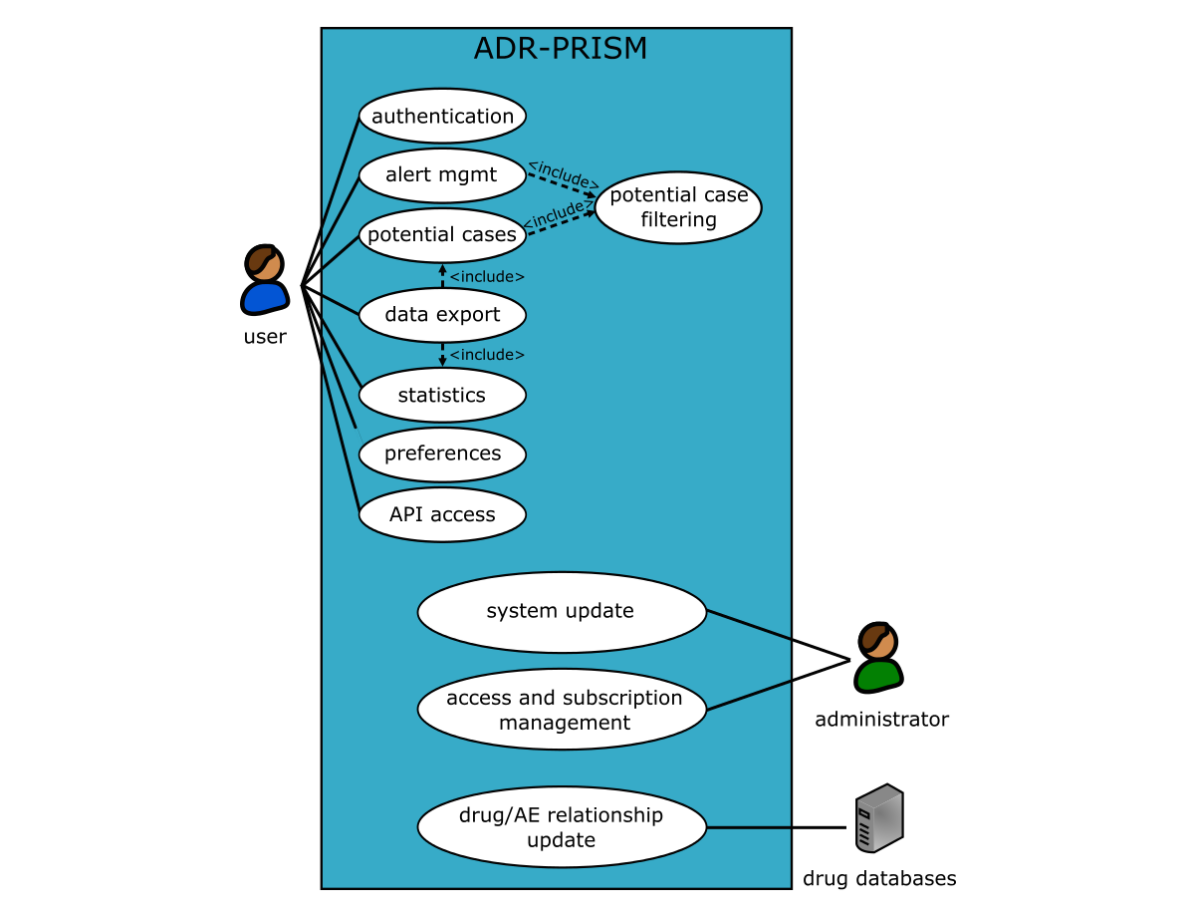
Use cases of the project.

A total of 11 use cases have been described: 8 for the user, 2 for the administrator, and 1 for the update of drug/ADR relationships. Use case diagrams are depicted in Figure 1. The user is a pharmacovigilance specialist who needs to review potential ADRs reported by patients in social media. The administrator is working in the back office of the ADR-PRISM platform in order to update the system and manage the user accounts.

### Identification and Processing of Relevant Information Within Web Pages

As a result of the annotation, we obtained lists of medical entities coming from the different terminologies that have been automatically converted into the Expert System Skill Cartridges format. No manual work was done at this stage, only recommendations on incongruities and ambiguities. The extracted entities were symptoms, medications, patients, and pathologies.

Relationships based on extraction rules give information on the occurrence of an ADR which can be absent (the treatment is effective and does not cause adverse reaction) or present (the treatment causes adverse reactions), as depicted in Table 5. To identify these reactions, the Skill Cartridge leverages the different thesauri and embeds specific extraction patterns and rules.

The extraction rules rely on trigger words coming from the terminologies and on semantic or syntactic patterns, which describe the syntactic phrases. A context rule expresses combinations relying on contextual triggers. A semantic rule consists of combinations of meaningful units of analysis (ie, concepts defined in previous rules). A syntactic rule consists of combinations of units relying on the syntactic structure and predication. Roles are associated to concepts depending on their function.

**Table 5:** Examples of drugs and adverse reactions extracted by the annotation.

Drug effects	Medical entity	Example
Present (ie, adverse reactions)	Drug: citalopram (Racine Pharma); Adverse reactions: insomnia, nausea (MedDRA^a^)	“*Citalopram* gave me the same *side effects* as setraline, *insomnia* and *nausea.*”
Absent	Drug: androcur (Racine Pharma); Adverse reactions: weight gain (MedDRA)	“My taking of *androcur* didn’t have *weight gain* as a consequence.”

^a^MedDRA: Medical Dictionary for Regulatory Activities.

### Robust and Evolutive Architecture

**Figure 2:**
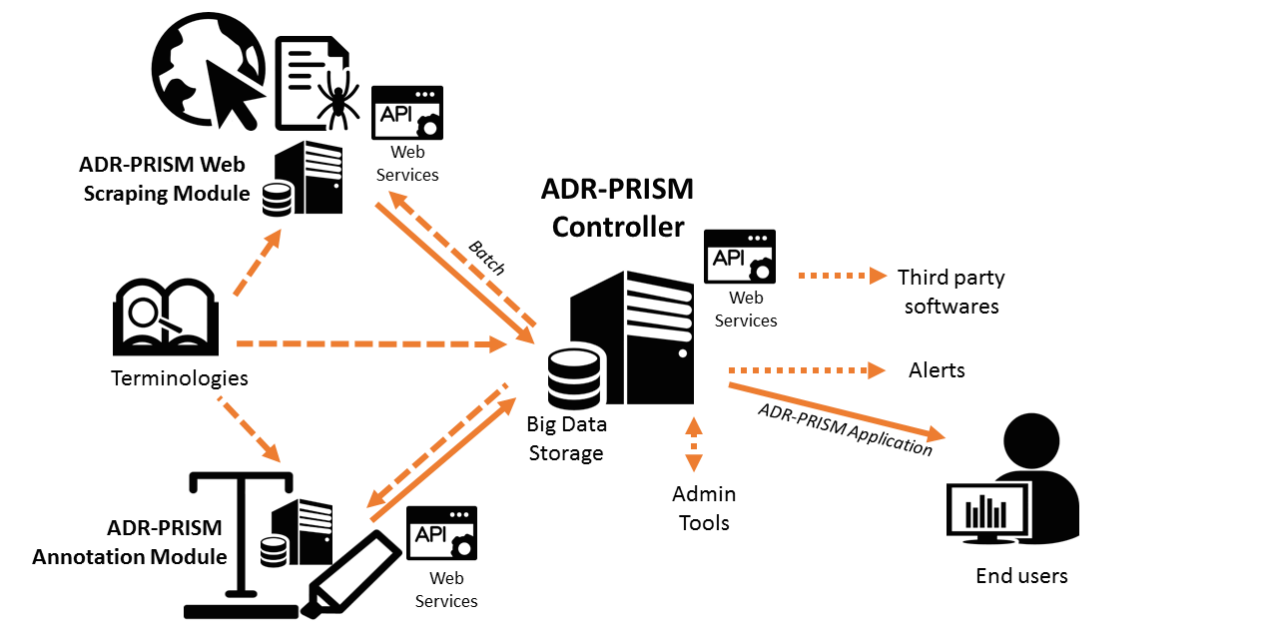
The technical architecture of the Adverse Drug Reactions from Patient Reports in Social Media project.

The ADR-PRISM information system fits well with the SaaS (software as a service) delivery model: it can be accessed by light clients such as Web browsers and is interoperable through the Web services it provides. A short overview of the ADR-PRISM information system is described in the technical architecture schema depicted in Figure 2.

Technically speaking, the ADR-PRISM component-based architecture is mainly composed of the following:

- Web scraping module: this crawling server provides Web services to launch the crawling of new posts of a given website from a given date and recover the cleaned content. Both Simple Object Access Protocol and Representational State Transfer are supported. The administrators of the crawling server punctually supply the crawling tool with useful terminology data at this stage (names of the roots of medicinal products, for example). A specific parsing program is written for the filtering and cleaning process of each website. Inheritance is of course used for common functions. In addition to the post’s textual content, metadata linked to the post are also delivered by this service. The result of the client call to the ADR-PRISM Web scraping service by the ADR-PRISM controller is stored in the common database for further purposes, such as text annotation service calls.
- Text annotation module: the Luxid Annotation server provides Web services to launch the automatic annotation of posts passed as parameter. The administrators of the annotation server punctually supply it with data from the terminology repositories to update the Skill Cartridges. After the call to this annotation service by the controller, posts are labeled with known controlled vocabulary concepts (provided by terminology servers).
- Terminology standards: exports are made from the Vidal terminology servers and HeTOP [20]. The HeTOP portal includes more than 69 health terminologies and provides services to export those data with standard formats. These exports are loaded in the modules requiring terminological information scraping and annotation tools but also the common database (data source for the graphical user interface). Terminological data can be used in several steps of the ADR-PRISM information system: to preselect posts, to create a Skill Cartridge, or for data mining or querying purposes.
- Common database: this serves to host the data necessary to the ADR-PRISM application server, whether batch process (controller jobs) or graphical user interface. This database especially contains monitored URLs, forums related metadata (name, last analysis dates, etc), crawling results (threads and posts to annotate and related metadata), annotation results, terminological data, and necessary information for the user interface (users, alerts, statistics, etc). The chosen solution for big data storage is PostgreSQL, which offers the best compromise for ADR-PRISM problematics—besides its proven reliability, it especially combines horizontal scalability features and advanced querying capabilities.
- Controller: this application server is a client of the data collection and annotation services. Batch processes are initiated at regular intervals: they consult the common database to respectively identify the URLs to process and posts to annotate, after which they query the required services and store the response in the database. Given that this batch process regularly launches the scraping, a lag can occur with the initial data in proportion of the time interval between each passage. The server also hosts the graphical interface Web application and the administration application. It also sends alerts to users according to their preferences and communicates through the interoperable Web services that allow access to the ADR-PRISM system-collected data for third-party applications.

## Discussion

### Principal Findings

In this paper we presented 5 major challenges that must be overcome to exploit patient posts as a complementary knowledge source for pharmacovigilance in professional settings such as drug regulatory agencies and the pharmaceutical industry. We expect that our proposals add new contributions to the field by taking into account some aspects that make sense when a software application processing large volumes of posts to support pharmacovigilance intends to be applied in an operational way: (1) developing a scoring method to evaluate the quality of information of social media, (2) guaranteeing safe application of data privacy thanks to data minimization and restricted control of access, (3) analyzing the pharmacovigilance expert’s expectations to figure best practices to integrate this complementary knowledge source consisting of selected posts on putative ADRs in current pharmacovigilance process, (4) developing a data dictionary, pattern-based matching, and natural language processing techniques for the efficient identification of relevant information on ADRs within Web pages, and (5) implementing a robust and evolutive architecture that allows storing big data from medical Web forums and access by third-party applications.

### Comparison With Prior Work

#### Monitoring Tools for Drug Safety Using Patient Posts

Existing tools already exist for analyzing patient posts about drug use on the Internet like Treato IQ [26]. This tool aims to assess what patients say about their treatment (ie, the patient voice). This professional platform gives health and life science industries insight into what patients think about their brand. Contrary to the ADR-PRISM project, the Treato main objective is more related to eReputation (the perception that Web users have about a brand, a company, or employees), and the platform is for pharmaceutical marketers and health care advertising and marketing agencies. ADR-PRISM aims to give regulatory agencies, pharmaceutical companies, and health care professionals insight about ADRs in social media, and its main objective is related to drug safety. Web Recognizing Adverse Drug Reactions (Web-RADR) is a European project that aims to recommend policies, frameworks, tools, and methodologies for the analysis of ADRs reported by patients in social media [27], but the consortium has not yet reported these recommendations.

Several points were partially or insufficiently documented by previous authors such as evaluation of social media, data privacy, and pharmacovigilance requirements. The technical architecture was usually implemented in previous works to allow a retrospective analysis based on a single extraction and did not support near real-time indexing, storage of big data, or interoperability with third-party applications that are desirable features in operational settings.

#### Variable Quality of Information on Social Media

It is expected that different social media may present different levels of quality of information about ADR descriptions. However, previous studies did not address issues related to measuring the quality of information of these social media (eg, selecting a Web forum containing threads about cancer in order to extract information on anticancer drugs [28]). Some tools exist to rate websites [17], but none of them has been designed to evaluate social media. We identified 21 criteria for evaluating social media in order to select those that present the most informative and relevant content to support pharmacovigilance processes with extraction and analysis of potentially interesting descriptions of ADRs.

#### Guarantee of Data Privacy

Data privacy was seldom discussed as an issue when extracting information from social media. A recent systematic review about attitudes toward ethics of researchers who explore data in social media showed very different approaches on whether social media should be seen as public or private space [29]. Indeed, one may believe that privacy does not apply online because information becomes public once it is posted, which allows access and use for research purposes, and that users should be responsible for preserving anonymity the way they manage their identity. Additionally, seeking consent from forums users is usually not feasible. There are still ethical issues in using social media for the extraction of new medical knowledge, the most important being that each patient posting on a forum should be guaranteed to keep his anonymity [30]. Considering data extraction for pharmacovigilance, anonymization was the main solution to guarantee data privacy such as implemented by Benton et al [28]. However, despite the anonymization process, patients may be reidentified a posteriori as described by Zimmer [31]; his analysis of a public release of anonymized Facebook data shows that a special mechanism should be implemented to limit access only to authorized personnel. This is the reason why we designed procedures to guarantee that only data going through a step of data minimization should be accessible for analysis by registered end users, but we kept raw data accessible in very specific circumstances to allow contacting the patient if drug withdrawal was required for safety reasons.

#### Response to Pharmacovigilance Expert Expectations

In order that analysis of data retrieved from patient posts could be implemented in current pharmacovigilance processes, it is desirable to first describe these processes and to identify the pharmacovigilance evaluator requirements and expectations. We could identify these requirements in previous work only from a broad scope (ie, discussing issues related to underreporting and the need for a complementary knowledge source). We believe that previous works have insufficiently taken into account the way pharmacovigilance experts are working and how the new suggested methods should be complementary with current pharmacovigilance processes. We explored the pharmacovigilance experts' expectations and identified several issues that should be addressed by designing and implementing new functionalities supported by appropriate graphical user interface.

#### Identification and Processing of Relevant Information Within Web Pages

While other challenges were seldom described in previous work, identification of drugs and ADRs in Web pages was the main objective for studies focusing on extraction of relevant information from Web forums for pharmacovigilance. On one hand, several publications [28,32-35] only deal with cooccurrences of drug names and adverse reactions in messages from social media. On the other hand, some studies [18,36-41] went further and attempted to qualify the relationship between occurrences of drugs and adverse reactions. The following methods have been applied to patient posts in social media: specialized dictionaries [28,32,34,36-39,42-43], pattern-based matching [38], machine learning [18,37,42-44].

Our approach enforces best practices based on specialized dictionaries, pattern-based matching, and natural language processing. We selected 4 terminologies to feed the terminological services. One limit is that the Skill Cartridges are still in development, which prevents us from conducting a formal study in order to evaluate their performance and compare them with other approaches already described in previous work. However, results obtained by the current version of the Skill Cartridges are encouraging.

#### Robust and Evolutive Architecture

The number of posts extracted from forums was variable in previous work, ranging from small samples (<20,000 posts [32,40]) to larger extractions (over one million posts [28,33]). But none had to face big data issues except Bian et al [18], who collected 2 billion Tweets. We found only one article discussing specific technologies for storing large numbers of tweets with Hadoop and Apache Hive [45].

Moreover, these applications were based on a retrospective extraction of patient posts and did not take into account the requirement to automatically collect new posts on a regular basis. We consider that extraction should be available through a near real-time indexing technique to allow analysis of patient posts in pharmacovigilance daily routine.

The barriers we had to overcome were (1) making the system robust and reliable (accelerating and securing Web services to be able to add and to rapidly evolve services and technological solutions without putting risk on the platform) and (2) opening the system to the outside to offer native interoperability by advancing from the software model to a large modular service platform. The ADR-PRISM platform supports storage of big data and access to third-party software through Web services and is based on a modular architecture consisting of different components to handle terminological, scraping, and annotation services.

### Perspectives

Although the part of posts that describe personal experiences of an ADR may correspond to a very small subset of the available posts on the Internet, the large number of data sharing in social media makes such data valuable material for pharmacovigilance [46]. Analyzing patient narratives in discussion forums or blogs is important to explore patient-centered issues that may not be detected in other existing sources generated by health care professionals [47].

In the early stages of the ADR-PRISM project, we studied the requirements of final users and defined a technical architecture that allows the efficient extraction and exploitation of ADRs from social media. The consortium approach for guarantee of data privacy may change depending on new regulatory developments. In particular, the European general regulations on data protection will be taken into account when they apply. As the 21 criteria selected for evaluating the quality of social media are independent from the application domain, additional research would be necessary to determine those that allow identification of the most informative and relevant content to support pharmacovigilance processes with extraction and analysis of potentially interesting descriptions of ADRs.

Perspectives of annotation in patient posts are to evaluate the performance of the annotation module compared to previous work and to take into account specific issues related to lay language [28,32,36,40,44]. An additional thesaurus containing patient language must be created in order to normalize the vocabulary found in the messages so that it can be recognized by medical reference thesauri. We will take into account the number of words between the detected drug and event as recent evidence shows that such distance can be used for identifying false positives and filter events that are likely to be ADRs [48].

A second prototype is being elaborated that provides a user interface for browsing documents and performing direct searches with the following functionalities: semantic facets that enable multidimensional navigation within documents and highlighting and definition of concepts within the text.

In the next steps, we will evaluate posts identified by the system in social media in order to clarify the interest and relevance of such an approach to improve conventional pharmacovigilance processes based on spontaneous reporting. Any pharmacovigilance system will be enabled to use the ADR-PRISM platform through Web services by selecting a specific drug, drug reaction, class of drug, or type of ADR. All potential ADRs will be extracted and filtered; a triage function at intake will help to identify case reports that have high potential, regarding the drug implicated or the reaction described, to enable a proactive surveillance. For example, the seriousness of the reaction will be assessed, adverse reactions concerning specific populations (pregnant women, etc) will be identified, and pharmacovigilance signals will be detected. Specific drug use may also be identified to detect possible cases of nonmedical use of pharmaceutical products [49].

Regarding languages processed by the ADR-PRISM system, the application scope of the project is focused on French and English data. But more languages could be added in the future (terminological data are multilingual; for example, MeSH or MedDRA are available respectively in 16 and 11 languages in HeTOP [21]). Other applications such as identification of counterfeiting and eReputation analysis for pharmaceutical companies should also be considered.

## Appendix

Multimedia Appendix 1 List of the 21 selected criteria for the evaluation grid.

## Abbreviations

ADR: adverse drug reaction
ADR-PRISM: Adverse Drug Reactions from Patient Reports in Social Media
ATC: Anatomical Therapeutic Chemical Classification System
CISMeF: Catalogue et Index des Sites Médicaux de langue Française
CNIL: Commission Nationale de l'Informatique et des Libertés
HeTOP: Health Terminology/Ontology Portal
MedDRA: Medical Dictionary for Regulatory Activities
SaaS: Software as a Service
Web-RADR: Web Recognizing Adverse Drug Reactions
