# Supplementary material for: The Adverse Drug Reactions from Patient Reports in Social Media Project: Five Major Challenges to Overcome to Operationalize Analysis and Efficiently Support Pharmacovigilance Process
Source: JMIR Res Protoc. 2017 Sep 21;6(9):e179. doi: 10.2196/resprot.6463 (PMC5629348; doi:10.2196/resprot.6463)
Supplement: Multimedia Appendix 1 [file resprot_v6i9e179_app1.pdf]

Appendix 1: List of the 21 selected criteria for the evaluation grid

- Each message is posted under a pseudonym.
- Context: sponsored or independent web site (moreover, advertisements should not be mistaken for content).
- Conflict of interest (associated with the web site).
- Subjectivity, bias (associated with the web site).
- Date of the last modification of the post.
- Existing editorial board.
- Presence of an administrator (Heath professional? Full-time administrator?).
- Writing quality (minimal SMS language and abbreviations; correct punctuation).
- Truthfulness of the messages (no “internet trolls”).
- Organization of the forum (Are the topics well located? If not, are they moved by the moderator?).
- Quality of the embedded search engine (accessibility, usability and exhaustivity).
- Presence in most search engines.
- Exhaustivity of the necessary information the user has to give to subscribe.
- Exhaustivity of the information addable by the user to his profile.
- Access to profiles with different security layers (with or without subscription).
- “Like” system: existence and pertinence (Is it possible to “like” without subscribing? Is there a dislike or a downvote system?).
- Users' qualification pertinence.
- Alert system to help moderators identify counterfeit posts.
- Number of messages over a certain period; comparison, on the same topic, with Doctissimo.
- Number of new topics over a certain period.
- Quality of the assistance (Administration, FAQ,…).
